# Prevalence and classification of anemia among children in Sudan: a systematic review and meta-analysis

**DOI:** 10.25122/jml-2023-0441

**Published:** 2024-08

**Authors:** Mubarak Ibrahim Idriss, Abd Alhadi Adam Hussein, Ali Mussa, Elshazali Widaa Ali, Ibrahim Khider Ibrahim, GadAllah Modawe, Ezeldine Abdalhabib, Muhammad Saboor, Khalid Hajissa

**Affiliations:** 1 Department of Hematology, Faculty of Medicine and Health Sciences, Kassala University, Kassala, Sudan; 2 Department of Hematology, School of Medical Sciences, Universiti Sains Malaysia, Kubang Kerian, Kota Bharu, Kelantan, Malaysia; 3 Department of Biology, Faculty of Education, Omdurman Islamic University, Omdurman, Sudan; 4 Center for Global Health Research, Saveetha Medical College and Hospitals, Saveetha Institute of Medical and Technical Sciences, Chennai, India; 5 Department of Medical Laboratory Science, College of Applied Medical Science, University of Bisha, Bisha, Saudi Arabia; 6 Department of Hematology, Faculty of Medical Laboratory Sciences, Al Neelain University, Khartoum, Sudan; 7 Department of Biochemistry, Faculty of Medicine and Health Science, Omdurman Islamic University, Omdurman, Sudan; 8 Department of Clinical Laboratory Sciences, College of Applied Medical Sciences, Jouf University, Sakakah, Saudi Arabia; 9 Department of Medical Laboratory Sciences, College of Health Science, University of Sharjah, Sharjah, United Arab Emirates; 10 Department of Zoology, Faculty of Science and Technology, Omdurman Islamic University, Omdurman, Sudan

**Keywords:** anemia, iron deficiency anemia, children, Sudan, systematic review, meta-analysis

## Abstract

Anemia remains a significant public health concern, particularly in developing countries like Sudan, where children are particularly vulnerable to its associated health implications. This study aimed to systematically assess the prevalence of anemia among Sudanese children. We conducted a comprehensive literature search in August 2021 and updated it in December 2022 to identify studies on anemia among Sudanese children. We searched databases including PubMed, Scopus, Science Direct, and Google Scholar. Studies eligible for inclusion in this systematic review and meta-analysis (SRMA) reported data to calculate anemia prevalence in children using WHO criteria. We performed meta-analysis using R software with metaprop and metafor packages. Using a random effects model, we estimated the pooled prevalence of anemia among 8006 Sudanese children to be 53.5% (95% CI, 36.6–70.4%). Subgroup analysis revealed that 62.7% of the children had unclassified anemia, 39.1% had iron deficiency anemia, and 8.7% had sickle cell anemia. Over a 21-year period, childhood anemia in Sudan showed an increasing trend, rising from 40.7% (1999–2009) to 55.1% (2010–2015) and 58.1% (2016–2020). The findings indicate a significantly higher prevalence of anemia among Sudanese children compared to many other countries, highlighting the need for proactive measures to prevent and control anemia in this population.

## INTRODUCTION

Anemia is a significant global public health problem affecting both developed and developing countries, including Sudan [1]. According to the World Health Organization (WHO), anemia is defined as a condition in which the number of red blood cells or their oxygen-carrying capacity is insufficient to meet physiologic needs that vary with age, gender, altitude, smoking status, and pregnancy status [2]. Children, pregnant women, and women of childbearing age are particularly vulnerable to anemia, which is associated with poor cognitive development, increased mortality, reduced immunity, and stunted growth during childhood [3–5]. Long-term effects of childhood anemia can impair social interaction and work productivity later in life, impacting individual quality of life and national socio-economic development [6,7].

In 2019, the World Health Organization (WHO) reported that 269 million children (39.8%) aged 6-59 months were affected by anemia worldwide, with the highest prevalence reported in Africa [8]. Understanding the underlying etiology of anemia among children is crucial for making informed decisions to reduce its prevalence. Anemia has a multifactorial etiology [9], but two main factors stand out: micronutrient deficiency [10] and parasitic infections. Iron deficiency is the leading cause of anemia [11], while malaria is the most common parasitic infection associated with anemia. Additionally, it has been linked to other factors such as age, gender, race, lower maternal education, and a lower household wealth index [12,13].

In Sudan, there is a notable scarcity of national-level surveys that provide data on the prevalence of anemia among its population. The most recent nationally representative data on childhood anemia in Sudan dates back to 1995, when a survey covered only a few states, revealing a prevalence of over 80 % [14]. Most available information on anemia in children comes from small-scale studies and healthcare reports, which show significant disparities in anemia prevalence. The limitations of these studies hinder their use as a baseline for monitoring anemia trends.

Despite efforts by the Federal Ministry of Health to implement various anti-anemia interventions, including promoting dietary diversity, fortification, improving hygiene, and infection control, there remains a pressing need to comprehensively review the existing literature regarding anemia prevalence among children in Sudan [15]. To our knowledge, no systemic review or meta-analysis of anemia prevalence among children in the country has been conducted. Accordingly, the current study is the first to summarize and critically analyze the available literature on the prevalence of anemia among children in Sudan, highlighting health risks associated with childhood anemia, the considerable variation in published studies on this topic, and the necessity of accurate prevalence estimation for effective prevention and management strategies.

## MATERIAL AND METHODS

This study adhered to the guidelines outlined in the Preferred Reporting Items for Systematic Reviews and Meta-analyses (PRISMA) [16].

### Search strategy

A comprehensive literature search was conducted on August 15, 2021, with an update performed on December 2, 2022, to identify relevant studies concerning anemia among children in Sudan. Databases, including PubMed, Scopus, Science Direct, and Google Scholar were searched. Reference lists of included articles were also reviewed to ensure comprehensive coverage. The detailed search strategy for all databases is available in Table S2.

### Inclusion and exclusion criteria

Studies were included in this systematic review and meta-analysis if they met the following criteria: conducted in Sudan, reported or provided adequate data to calculate the prevalence of anemia in children using WHO criteria, and had no restrictions based on age, gender, language, or the period in which the studies were conducted or published. Exclusion criteria were studies conducted among children with comorbidities such as HIV/AIDS, renal disease, and other medical or surgical conditions, as well as non-original papers like review articles, case reports, case studies, and studies with only abstracts.

### Data management and study selection

All identified studies were managed using EndNote (Clarivate Analytics). Duplicate records were removed, and the remaining studies were screened by title and abstract. The full texts of potentially eligible studies were then reviewed. Two authors (KH and MII) independently evaluated the eligibility of the studies based on predefined criteria. Any disagreements were resolved through discussion with a third author (AM) until a consensus was achieved.

### Data extraction

Two reviewers (KH and MII) independently extracted data from the included studies using a standardized form. To ensure accuracy and consistency, a third author (IKI) performed a secondary check. Extracted data included the first author’s last name, publication year, study design, enrollment period, region/province, and the distribution of gender and age in the study population.

### Quality assessment

The quality of the included studies was independently assessed by two authors (KH and AM) using the Joanna Briggs Institute (JBI) critical assessment checklist for cross-sectional studies [17]. The assessments were compared, and any discrepancies were resolved through consensus. Studies scoring over 70% were classified as low risk of bias (high quality), those scoring 50%-70% as moderate risk (moderate quality), and those scoring below 50% as high risk of bias [18,19].

### Data analysis

Meta-analysis was conducted using the metaprop codes within the meta package (version 4.15-1) and the metafor package (version 2.4-0) in R (version 3.6.3) with RStudio (version 1.3.1093). Pooled estimates of anemia prevalence among children and the 95 percent confidence intervals (CIs) were calculated using random-effects models employing the REML method. To assess study heterogeneity, both I2 statistics and Cochran's Q-test were utilized. An I2 statistic exceeding 75% and a significance level of less than 0.05 indicated significant heterogeneity. Prior to conducting significance testing with Egger's test, the funnel plot was visually inspected for any signs of publication bias. Furthermore, potential sources of heterogeneity were further explored through subgroup analysis, considering factors such as anemia type, publication year, region, study setting, and recruitment location.

## RESULTS

### Study selection

**Figure 1:**
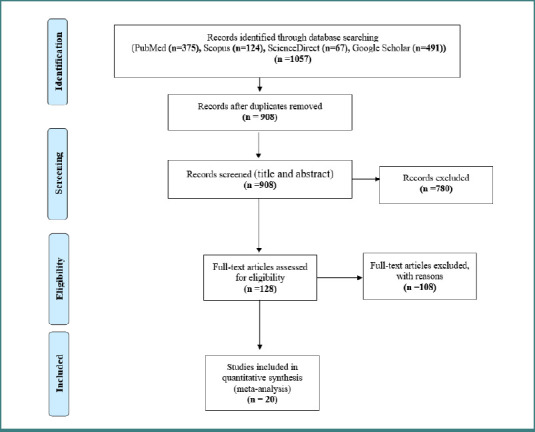
PRISMA flow diagram of literature search and study selection

A detailed flow diagram illustrating the screening and selection process of the studies is presented in Figure 1. Initially, a total of 1057 studies were identified from the four electronic databases. After removing duplicates (*n* = 149), the titles and abstracts of the remaining 908 studies were assessed, and 780 irrelevant studies were subsequently excluded based on our inclusion criterion. This left 128 studies eligible for full-text review. Only 20 studies met the eligibility criteria and were included in this systematic review and meta-analysis.

### Characteristics of included studies

Table 1 outlines the characteristics of the 20 studies included in this analysis. All the studies utilized a cross-sectional design, with sample sizes ranging from 100 to 3094 participants. The earliest study was conducted in 1999 [20], while the most recent took place in 2020 [21]. Geographically, 11 studies were carried out in the central region of Sudan, four in the Eastern region, three in the Northern region, one in the Western region, and one across multiple regions. Among the 20 studies, eight were conducted in school settings, another eight in community settings, and the remaining four were hospital-based. This systematic review and meta-analysis included data from 8006 children between the ages of 0 to 18 years.

**Table 1:** Major characteristics of the included studies

Author, year [Ref]	Study period	Study area	Recruitment location	Children	Age (Year)	Gender	Sample size	Type of anemia
Abdelrahim 2009 [21]	Sept. to Oct. 2008	New Halfa	School	School-age children	14-18	F	187	IDA, Folate and coper deficiency
Abdelrahman 2018 [22]	Jane 2014 to Feb. 2015	Wad Madani	Community	Preschool children	3-5	M and F	300	Unclassified
Adam 2019 [23]	Dec. 2017 to Aug. 2018	Alfasher	Hospital	Non Specific	0-18	M and F	400	Sickle cell anemia
Ahmed 2014 [24]	2014	River Nile state	Community	School-age children	6-7	M and F	124	IDA
Ahmed 2019 [25]	2013	Al Gadaref	Community	Preschool children	0.5-5	M and F	384	Unclassified
El Tayeb 2016 [26]	2014	Gezera state	School	School-age children	8-18	M	180	Unclassified
El-Hag 2016 [27]	June 2015 to Dec. 2015	Singa	Community	Preschool children	0-5	M and F	250	Unclassified
Elmardi 2020 [18]	Nov. 2016	18 states (multiple regions)	Community	Preschool children	0.5-5	M and F	3094	Unclassified
Eltieb 2018 [28]	2017	Wad Madani	Hospital	Preschool children	0-5	M and F	208	Unclassified
Gibla 2013 [29]	2013	Khartoum	Hospital	Preschool children	0.2-6	M and F	500	IDA
Habib Allah 2009 [20]	Nov. 2008 to May 2009	Khartoum	School	School-age children	6-18	M	100	IDA
Hussein 2014 [30]	2014	Northern Sudan	School	Preschool children	3-6	M and F	163	Unclassified
Ibrahim 2020 [31]	2020	Khartoum	Community	School-age children	13-18	M	100	Unclassified
Mahgoub 2019 [32]	2018	Gezera	Community	Preschool children	0-5	M and F	170	Unclassified

IDA, Iron Deficiency Anemia; M, male; F, female

### Quality assessment and publication bias

**Figure 2:**
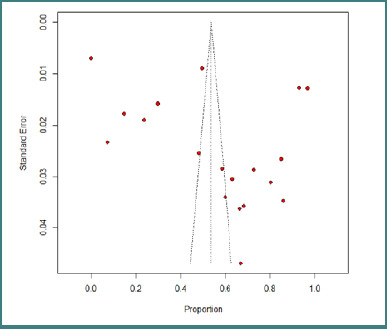
Funnel plot of overall prevalence of anemia among children in Sudan

The summary assessment of the quality of individual studies can be found in supplementary Table S1. In brief, the risk of bias was determined to be high in 11 (64.7%) studies, moderate in 28 (64.7%) studies, and low in 30 (64.7%) studies. The presence of publication bias is indicated by the asymmetrical funnel plot (Figure 2), and the bias was statistically confirmed by Egger’s test (*P* = 0.48 and < 0.0001).

### Prevalence of anemia

**Figure 3:**
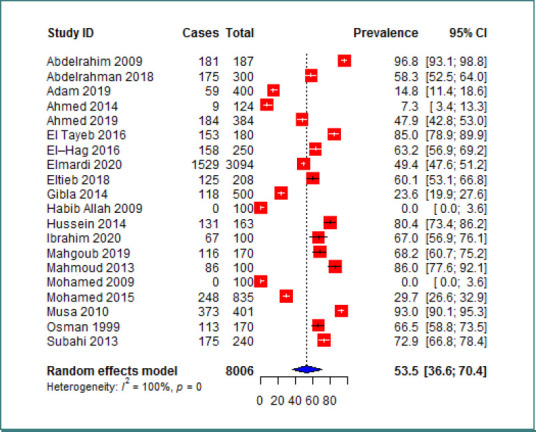
The overall prevalence of anemia among children in Sudan

The prevalence of anemia among Sudanese children had significant variation, ranging from 0%, as reported in two studies conducted in Khartoum, Central Sudan [22,23], to 96.8 %, as reported in New Halfa, Eastern Sudan [24]. Employing a random-effects model, the overall pooled prevalence of anemia among the 8006 children recruited in the 20 included studies was estimated at 53.5% (95% CI, 36.6–70.4%), with substantial heterogeneity observed between the included studies (*I*^2^= 99.8%, *P* < 0.0001) (Figure 3).

### Subgroup analysis

Subgroup analysis was performed to explore the potential sources of heterogeneity. Table 2 and Figure S1 display the prevalence of anemia among Sudanese children across various subgroups. The pooled estimates based on anemia type showed that 62.7 % (95% CI, 48.9–76.4%) of the children reported in 11 studies had unclassified anemia, while 39.1% (95% CI, 15.1–62.4%) recruited in 7 studies had iron deficiency anemia (IDA), and 8.7% (95% CI, 0.0–20.4%) had sickle cell anemia. Additionally, folate, copper, and zinc deficiencies were estimated to be 68.9%, 5.9% and 9.1%, respectively.

**Table 2:** Pooled prevalence of anemia in different subgroups

Subgroups	Prevalence [95% CIs] (%)	Number of studies analyzed	Total number of subjects	Heterogeneity	
				*I* ^2^	*P* value
**Total**	53.5 [36.6–70.4]	20	8006	99.8%	< 0.0001
**Type of anemia**					
**Unclassified anaemia**	62.7 [48.9–76.4]	11	6145	99.2%	< 0.0001
**Iron deficiency anemia**	39.1 [15.8–62.4]	7	1361	99.6%	< 0.0001
**Sickle cell anemia**	8.7 [0.0–20.4]	2	580	96.8%	< 0.0001
**Folate deficiency anemia**	68.9 [62.4–75.6]	1	187	NA	NA
**Copper deficiency**	5.9 [2.5–9.3]	1	187	NA	NA
**Zinc deficiency**	9.1[4.9–13.2]	1	187	NA	NA
**Publication year**					
**Year 1999-2009**	40.7 [ 0.0–81.7]	4	557	99.9%	<0.0001
**Year 2010-2015**	55.1 [30.2–79.9]	8	2747	99.7%	<0.0001
**Year 2016-2020**	58.1 [42.9–73.4]	8	4702	98.9%	< 0.0001
**Different regions of Sudan**					
**Eastern Sudan**	81.0 [63.1–98.9]	4	1072	99.0%	< 0.0001
**Central Sudan**	51.2 [33.3–69.1]	11	2318	99.7%	<0.0001
**Western Sudan**	14.7 [11.3–18.2]	1	400	NA	NA
**Northern Sudan**	39.0 [ 4.8–73.2]	3	1122	99.4%	< 0.0001
**Multiple regions of Sudan**	49.4 [47.7–51.2]	1	30	NA	NA
**Study setting**					
**Urban**	50.9 [26.4–75.3]	12	3030	99.9%	<0.0001
**Rural**	57.2 [30.9–83.4]	6	1712	99.4%	< 0.0001
**Both**	49.4 [47.7–51.2]	1	3094	NA	NA
**Recruitment location**					
**School**	56.4 [24.5–88.2]	8	2136	99.9%	0
**Community**	54.1 [40.7–67.6]	8	4662	98.5%	< 0.0001
**Hospital**	45.9 [17.8–74.15]	4	1208	99.3%	< 0.0001

The assessment of childhood anemia in Sudan over a 21-year period revealed a clear upward trend. The prevalence of anemia in Sudanese children increased from 40.7% (95% CI, 0.0–81.7%) during 1999–2009 to 55.1% (95% CI, 30.2–79.9%) during 2010–2015 and further to 58.1% (95% CI, 42.9–73.4%) in the subsequent five years (2016–2020). In terms of the regions in Sudan, the highest rate of anemia among Sudanese children was observed in Eastern Sudan (81.0%; 95% CI, 63.1–98.9%), followed by Central Sudan (51.2%; 95% CI, 33.3–69.1%), Northern Sudan (39.0%; 95% CI, 4.8–73.2%), and Western Sudan (14.7%; 95% CI, 11.3–18.2%). Meanwhile, studies conducted in multiple regions of Sudan revealed an overall prevalence of 49.4% (95% CI, 47.7–51.2%). In subgroup analysis, the prevalence of anemia among children in rural areas was 57.2% (95% CI, 30.9–83.4%), which was higher than that among urban areas, at 50.9% (95% CI, 8.8–54.4%). When estimating anemia prevalence based on the recruitment site, it was found to be higher in studies conducted at the school level, at 56.4% (95% CI, 24.5–88.2%), compared to 54.1% (95% CI, 40.7–67.6%) in community-based studies and 45.9% (95% CI, 17.8–74.15%) in hospital-based studies, respectively.

## DISCUSSION

In this systematic review and meta-analysis, we examined the pooled prevalence of anemia among children in Sudan for the first time. Our analysis, which encompassed data from 20 observational studies involving 8006 Sudanese children, revealed a pooled prevalence of anemia at 53.5%. Notably, the prevalence of anemia among Sudanese children exhibited significant variation across the included studies. As depicted in Figure 3, the highest and lowest anemia prevalence rates were reported in studies conducted in Khartoum, Central Sudan [22,23] and in New Halfa, Eastern Sudan (96.8 %), respectively [24]. Compared to previous research, the findings of this study indicated a higher overall prevalence of anemia among Sudanese children. For example, an SRMA conducted by Tezera *et al*. [25] reported a pooled prevalence of 23% among Ethiopian children. Furthermore, a study involving 1,700 Iranian children under the age of six found that 18.2% of them had IDA [26]. However, our result was lower than the prevalence reported in several studies conducted in Ghana [27] and the estimated 72% prevalence in Western and Central African countries [28]. The substantial variation in anemia prevalence among children is not unexpected and may be attributed to a range of factors, including, differences in study participants, sample sizes, sociodemographic characteristics, dietary habits, as well as variations in infection and disease rates.

A subgroup analysis was performed based on the type of anemia, revealing that the most prevalent type was unclassified (general) anemia, reported in 11 studies, with a prevalence rate of 62.7%. Additionally, 39.1% of the children recruited in seven studies were found to have IDA, while 8.7% were diagnosed with sickle cell anemia in two studies. Notably, iron deficiency is the leading cause of nutritional anemia worldwide, particularly in developing countries. However, it is worth mentioning that IDA is a relatively manageable condition in developed countries. Compared to previous meta-analyses, our study reported a considerably higher prevalence of IDA. In contrast to the higher prevalence of IDA observed in our study, Bangladesh and Iran reported a lower prevalence among children and adolescents, at 13.6% and 13.9%, respectively [29,30]. This variation could be attributed to several factors, including differences in healthcare infrastructure, nutritional practices, and the prevalence of specific health conditions. It is important to note that the higher prevalence of infectious and chronic diseases, hemoglobinopathies, and lead poisoning in developing countries such as Sudan can indeed contribute to the increased complexity and prevalence of IDA. These factors can have a significant impact on anemia rates in such regions.

The results of the meta-analysis highlight a significant variation in the prevalence of childhood anemia across different regions of Sudan. Eastern Sudan reported the highest anemia prevalence among children (81.0%), followed by Central Sudan (51.2%), Northern Sudan (39.0%), and Western Sudan (14.7%). It is worth noting that a previous meta-analysis carried out in Sudan [31] also identified Eastern Sudan as having the highest rate of anemia. However, it remains uncertain whether children in this region are more anemic than children in other parts of the country. The observed disparities in anemia rates among children from various Sudanese regions can likely be attributed to differences in sociodemographic factors, economic conditions, and dietary practices.

As indicated in Table 1, there were no significant differences in the overall prevalence of anemia when considering the recruitment site of the children. The results of the analysis from eight school-based studies and eight community-based studies yielded prevalence rates of 56.4% and 54.1%, respectively. Interestingly, in contrast to previous meta-analyses [32], the rate of childhood anemia in hospital-based studies was slightly lower (45.9%) than in school and community-based studies. Generally, the pooled estimate in a hospital setting tends to be higher than that in the community-based setting, possibly due to individuals in the hospital being affected by specific diseases that can influence hemoglobin levels. However, in this SRMA, the lower estimate in hospital-based studies may be attributed to the limited number of studies conducted in hospitals compared to other settings.

The assessment of childhood anemia in Sudan over a 21-year period has revealed a clear and concerning upward trend. The prevalence of anemia among Sudanese children increased from 40.7 % in 1999–2009 to 55.1% in 2010–2015 and further to 58.1% in the subsequent five years (2016–2020). This systematic review underscores that the prevalence of anemia among Sudanese children is notably higher than in many other countries worldwide. Considering that children constitute one of the most vulnerable populations and are at a high risk of anemia, the findings of this study have direct implications for anemia management in Sudan. Immediate preventive measures should be considered and implemented. These measures could include fortifying complementary foods with iron-containing micronutrient powders and the timely prevention and treatment of parasitic and infectious diseases. Addressing childhood anemia is crucial to improving the health and well-being of Sudanese children and reducing the long-term consequences associated with this condition.

The key strength of this SRMA lies in its pioneering effort to offer a comprehensive estimation of anemia among Sudanese children. However, the study does have certain limitations that should be considered. One notable limitation is that the included studies did not cover the entire country, and a limited number of papers were published in some regions. Consequently, the estimated prevalence of anemia may not fully represent the actual status of childhood anemia across the entire country. Moreover, the results from some regions may be slightly biased due to the limited data available. Despite the limitations, this SRMA serves as an important starting point in understanding the prevalence of childhood anemia in Sudan. It highlights the need for more extensive and representative research in the future to provide a more accurate and comprehensive picture of the anemia situation among Sudanese children.

## CONCLUSION

In conclusion, the prevalence of anemia remains a significant public health concern among Sudanese children. Therefore, there is a pressing need for the implementation, expansion, and careful monitoring of national nutritional intervention programs to improve the overall status of anemia. Furthermore, it is essential to conduct additional studies across Sudan to determine the true prevalence of anemia among Sudanese children accurately. The number of published studies is extremely limited, with no studies published in the last two years. Expanding the body of research in this area will contribute to a more comprehensive understanding of the anemia situation and facilitate the development of targeted interventions to address this critical health issue among Sudanese children.
